# Supplementary material for: Predation and fragmentation portrayed in the statistical structure of prey time series
Source: BMC Ecol. 2009 May 6;9:10. doi: 10.1186/1472-6785-9-10 (PMC2689204; doi:10.1186/1472-6785-9-10)
Supplement: Additional file 2 — Voles and related classes ODDox Documentation. ODDox documentation of the agent-based model (ALMaSS) applied by Hendrichsen et al. The documentation is started by activating main.html. [file 1472-6785-9-10-S2.zip › Vole_ODDox/_population_manager_8cpp.html]

ALMaSS ODDox: PopulationManager.cpp File Reference

- Main Page
- Related Pages
- Classes
- Files

# PopulationManager.cpp File Reference

---

## Detailed Description

**PopulationManager.cpp This is the code file for the population manager and associated classes**   

by Chris J. Topping   
Version of 23rd July 2003   
All rights reserved.   
  
With additions as noted in:   
January 2008   
Doxygen formatted comments in May 2008   

`#include <vector>`  
`#include <algorithm>`  
`#include <iostream>`  
`#include <fstream>`  
`#include "ls.h"`  
`#include "PopulationManager.h"`  

|  |  |
| --- | --- |
|  |  |
| Classes | |
| class | CompareState |
|  | Function to compare to TAnimal's Current behavioural state. More... |
| class | CompareX |
|  | Function to compare to TAnimal's m\_Location\_x. More... |
| class | CompareY |
|  | Function to compare to TAnimal's m\_Location\_y. More... |
| Defines | |
| #define | \_CRT\_SECURE\_NO\_DEPRECATE |
| #define | \_CRTDBG\_MAP\_ALLOC |
| Functions | |
| CfgInt | cfg\_CatastropheEventStartYear ("PM\_CATASTROPHEEVENTSTARTYEAR", CFG\_CUSTOM, 99999) |
| CfgInt | cfg\_CIPEGridOutput\_day ("G\_CIPEGRIDOUTPUT\_DAY", CFG\_CUSTOM, 1) |
| CfgInt | cfg\_CIPEGridOutput\_day\_b ("G\_CIPEGRIDOUTPUT\_DAY\_B", CFG\_CUSTOM, 270) |
| CfgStr | cfg\_CIPEGridOutput\_filename ("G\_CIPEGRIDOUTPUT\_FILENAME", CFG\_CUSTOM,"CIPEGridOutput.txt") |
| CfgStr | cfg\_CIPEGridOutput\_filenameB ("G\_CIPEGRIDOUTPUT\_FILENAME\_B", CFG\_CUSTOM,"CIPEGridOutputB.txt") |
| CfgInt | cfg\_CIPEGridOutput\_Interval ("G\_CIPEGRIDOUTPUT\_INTERVAL", CFG\_CUSTOM, 1) |
| CfgBool | cfg\_CIPEGridOutput\_used ("G\_CIPEGRIDOUTPUT\_USED", CFG\_CUSTOM, false) |
| static CfgInt | cfg\_DayInMonth ("PRB\_DAYINMONTH", CFG\_CUSTOM, 1) |
| CfgBool | cfg\_fixed\_random\_sequence ("G\_FIXEDRANDOMSEQUENCE", CFG\_CUSTOM, false) |
| CfgInt | cfg\_pm\_eventfrequency ("PM\_EVENTFREQUENCY", CFG\_CUSTOM, 0) |
| CfgInt | cfg\_pm\_eventsize ("PM\_EVENTSIZE", CFG\_CUSTOM, 100) |
| static CfgStr | cfg\_ReallyBigOutput\_filename ("G\_REALLYBIGOUTPUT\_FILENAME", CFG\_CUSTOM,"ReallyBigOutput.txt") |
| CfgBool | cfg\_ReallyBigOutput\_used ("G\_REALLYBIGOUTPUT\_USED", CFG\_CUSTOM, false) |
| static CfgInt | cfg\_RipleysOutput\_day ("G\_RIPLEYSOUTPUT\_DAY", CFG\_CUSTOM, 60) |
| static CfgStr | cfg\_RipleysOutput\_filename ("G\_RIPLEYSOUTPUT\_FILENAME", CFG\_CUSTOM,"RipleysOutput.txt") |
| static CfgInt | cfg\_RipleysOutput\_interval ("G\_RIPLEYSOUTPUT\_INTERVAL", CFG\_CUSTOM, 1) |
| CfgBool | cfg\_RipleysOutput\_used ("G\_RIPLEYSOUTPUT\_USED", CFG\_CUSTOM, true) |
| static CfgInt | cfg\_RipleysOutputFirstYear ("G\_RIPLEYSOUTPUT\_FIRSTYEAR", CFG\_CUSTOM, 1) |
| CfgBool | cfg\_RipleysOutputMonthly\_used ("G\_RIPLEYSOUTPUTMONTHLY\_USED", CFG\_CUSTOM, true) |
| static CfgBool | cfg\_UseEasyPop ("VOLE\_USEEASYPOP", CFG\_CUSTOM, false) |
| static CfgInt | cfg\_VoleCatastrophe\_interval ("VOLE\_CATASTROPHE\_I", CFG\_CUSTOM, 365 \*5) |
| static CfgInt | cfg\_VoleCatastrophe\_mortality ("VOLE\_CATASTROPHE\_M", CFG\_CUSTOM, 90) |
| static CfgBool | cfg\_VoleCatastrophe\_on ("VOLE\_CATASTROPHE\_ON", CFG\_CUSTOM, false) |
| Variables | |
| char | g\_str [255] |

---

## Define Documentation

#define \_CRT\_SECURE\_NO\_DEPRECATE

#define \_CRTDBG\_MAP\_ALLOC

---

## Function Documentation

|  |  |  |  |  |
| --- | --- | --- | --- | --- |
| CfgInt cfg\_CatastropheEventStartYear | ( | "PM\_CATASTROPHEEVENTSTARTYEAR" | , |  |
|  |  | CFG\_CUSTOM | , |  |
|  |  | 99999 |  |  |
|  | ) |  |  |  |

Referenced by Population\_Manager::Run().

|  |  |  |  |  |
| --- | --- | --- | --- | --- |
| CfgInt cfg\_CIPEGridOutput\_day | ( | "G\_CIPEGRIDOUTPUT\_DAY" | , |  |
|  |  | CFG\_CUSTOM | , |  |
|  |  | 1 |  |  |
|  | ) |  |  |  |

Referenced by Population\_Manager::Run().

|  |  |  |  |  |
| --- | --- | --- | --- | --- |
| CfgInt cfg\_CIPEGridOutput\_day\_b | ( | "G\_CIPEGRIDOUTPUT\_DAY\_B" | , |  |
|  |  | CFG\_CUSTOM | , |  |
|  |  | 270 |  |  |
|  | ) |  |  |  |

Referenced by Population\_Manager::Run().

|  |  |  |  |  |
| --- | --- | --- | --- | --- |
| CfgStr cfg\_CIPEGridOutput\_filename | ( | "G\_CIPEGRIDOUTPUT\_FILENAME" | , |  |
|  |  | CFG\_CUSTOM | , |  |
|  |  | "CIPEGridOutput.txt" |  |  |
|  | ) |  |  |  |

Referenced by Population\_Manager::OpenTheCIPEGridOutputProbe().

|  |  |  |  |  |
| --- | --- | --- | --- | --- |
| CfgStr cfg\_CIPEGridOutput\_filenameB | ( | "G\_CIPEGRIDOUTPUT\_FILENAME\_B" | , |  |
|  |  | CFG\_CUSTOM | , |  |
|  |  | "CIPEGridOutputB.txt" |  |  |
|  | ) |  |  |  |

Referenced by Population\_Manager::OpenTheCIPEGridOutputProbe().

|  |  |  |  |  |
| --- | --- | --- | --- | --- |
| CfgInt cfg\_CIPEGridOutput\_Interval | ( | "G\_CIPEGRIDOUTPUT\_INTERVAL" | , |  |
|  |  | CFG\_CUSTOM | , |  |
|  |  | 1 |  |  |
|  | ) |  |  |  |

Referenced by Population\_Manager::Run().

|  |  |  |  |  |
| --- | --- | --- | --- | --- |
| CfgBool cfg\_CIPEGridOutput\_used | ( | "G\_CIPEGRIDOUTPUT\_USED" | , |  |
|  |  | CFG\_CUSTOM | , |  |
|  |  | false |  |  |
|  | ) |  |  |  |

|  |  |  |  |  |
| --- | --- | --- | --- | --- |
| static CfgInt cfg\_DayInMonth | ( | "PRB\_DAYINMONTH" | , |  |
|  |  | CFG\_CUSTOM | , |  |
|  |  | 1 |  |  |
|  | ) |  |  | `[static]` |

Referenced by Population\_Manager::BeginningOfMonth().

|  |  |  |  |  |
| --- | --- | --- | --- | --- |
| CfgBool cfg\_fixed\_random\_sequence | ( | "G\_FIXEDRANDOMSEQUENCE" | , |  |
|  |  | CFG\_CUSTOM | , |  |
|  |  | false |  |  |
|  | ) |  |  |  |

|  |  |  |  |  |
| --- | --- | --- | --- | --- |
| CfgInt cfg\_pm\_eventfrequency | ( | "PM\_EVENTFREQUENCY" | , |  |
|  |  | CFG\_CUSTOM | , |  |
|  |  | 0 |  |  |
|  | ) |  |  |  |

|  |  |  |  |  |
| --- | --- | --- | --- | --- |
| CfgInt cfg\_pm\_eventsize | ( | "PM\_EVENTSIZE" | , |  |
|  |  | CFG\_CUSTOM | , |  |
|  |  | 100 |  |  |
|  | ) |  |  |  |

|  |  |  |  |  |
| --- | --- | --- | --- | --- |
| static CfgStr cfg\_ReallyBigOutput\_filename | ( | "G\_REALLYBIGOUTPUT\_FILENAME" | , |  |
|  |  | CFG\_CUSTOM | , |  |
|  |  | "ReallyBigOutput.txt" |  |  |
|  | ) |  |  | `[static]` |

Referenced by Population\_Manager::OpenTheReallyBigProbe().

|  |  |  |  |  |
| --- | --- | --- | --- | --- |
| CfgBool cfg\_ReallyBigOutput\_used | ( | "G\_REALLYBIGOUTPUT\_USED" | , |  |
|  |  | CFG\_CUSTOM | , |  |
|  |  | false |  |  |
|  | ) |  |  |  |

|  |  |  |  |  |
| --- | --- | --- | --- | --- |
| static CfgInt cfg\_RipleysOutput\_day | ( | "G\_RIPLEYSOUTPUT\_DAY" | , |  |
|  |  | CFG\_CUSTOM | , |  |
|  |  | 60 |  |  |
|  | ) |  |  | `[static]` |

Referenced by Population\_Manager::Run().

|  |  |  |  |  |
| --- | --- | --- | --- | --- |
| static CfgStr cfg\_RipleysOutput\_filename | ( | "G\_RIPLEYSOUTPUT\_FILENAME" | , |  |
|  |  | CFG\_CUSTOM | , |  |
|  |  | "RipleysOutput.txt" |  |  |
|  | ) |  |  | `[static]` |

Referenced by Population\_Manager::OpenTheMonthlyRipleysOutputProbe(), and Population\_Manager::OpenTheRipleysOutputProbe().

|  |  |  |  |  |
| --- | --- | --- | --- | --- |
| static CfgInt cfg\_RipleysOutput\_interval | ( | "G\_RIPLEYSOUTPUT\_INTERVAL" | , |  |
|  |  | CFG\_CUSTOM | , |  |
|  |  | 1 |  |  |
|  | ) |  |  | `[static]` |

Referenced by Population\_Manager::Run().

|  |  |  |  |  |
| --- | --- | --- | --- | --- |
| CfgBool cfg\_RipleysOutput\_used | ( | "G\_RIPLEYSOUTPUT\_USED" | , |  |
|  |  | CFG\_CUSTOM | , |  |
|  |  | true |  |  |
|  | ) |  |  |  |

|  |  |  |  |  |
| --- | --- | --- | --- | --- |
| static CfgInt cfg\_RipleysOutputFirstYear | ( | "G\_RIPLEYSOUTPUT\_FIRSTYEAR" | , |  |
|  |  | CFG\_CUSTOM | , |  |
|  |  | 1 |  |  |
|  | ) |  |  | `[static]` |

Referenced by Population\_Manager::Run().

|  |  |  |  |  |
| --- | --- | --- | --- | --- |
| CfgBool cfg\_RipleysOutputMonthly\_used | ( | "G\_RIPLEYSOUTPUTMONTHLY\_USED" | , |  |
|  |  | CFG\_CUSTOM | , |  |
|  |  | true |  |  |
|  | ) |  |  |  |

Referenced by Population\_Manager::Population\_Manager(), Population\_Manager::Run(), and Population\_Manager::~Population\_Manager().

|  |  |  |  |  |
| --- | --- | --- | --- | --- |
| static CfgBool cfg\_UseEasyPop | ( | "VOLE\_USEEASYPOP" | , |  |
|  |  | CFG\_CUSTOM | , |  |
|  |  | false |  |  |
|  | ) |  |  | `[static]` |

Referenced by Population\_Manager::SpeciesSpecificReporting().

|  |  |  |  |  |
| --- | --- | --- | --- | --- |
| static CfgInt cfg\_VoleCatastrophe\_interval | ( | "VOLE\_CATASTROPHE\_I" | , |  |
|  |  | CFG\_CUSTOM | , |  |
|  |  | 365 \* | *5* |  |
|  | ) |  |  | `[static]` |

|  |  |  |  |  |
| --- | --- | --- | --- | --- |
| static CfgInt cfg\_VoleCatastrophe\_mortality | ( | "VOLE\_CATASTROPHE\_M" | , |  |
|  |  | CFG\_CUSTOM | , |  |
|  |  | 90 |  |  |
|  | ) |  |  | `[static]` |

|  |  |  |  |  |
| --- | --- | --- | --- | --- |
| static CfgBool cfg\_VoleCatastrophe\_on | ( | "VOLE\_CATASTROPHE\_ON" | , |  |
|  |  | CFG\_CUSTOM | , |  |
|  |  | false |  |  |
|  | ) |  |  | `[static]` |

---

## Variable Documentation

char g\_str[255]

Referenced by Population\_Manager::ProbeReport(), and Population\_Manager::SpeciesSpecificReporting().

---

Generated on Thu Jan 22 14:13:45 2009 for ALMaSS ODDox by 
 1.5.6
